# Honeybee (*Apis mellifera*) Venom Reinforces Viral Clearance during the Early Stage of Infection with Porcine Reproductive and Respiratory Syndrome Virus through the Up-Regulation of Th1-Specific Immune Responses

**DOI:** 10.3390/toxins7051837

**Published:** 2015-05-22

**Authors:** Jin-A Lee, Yun-Mi Kim, Pung-Mi Hyun, Jong-Woon Jeon, Jin-Kyu Park, Guk-Hyun Suh, Bock-Gie Jung, Bong-Joo Lee

**Affiliations:** 1 Department of Veterinary Infectious Diseases, College of Veterinary Medicine, Chonnam National University, Gwangju 500-757, Korea; E-Mails: dvmjina@gmail.com (J.-AL.); kyli75@hanmail.net (Y.-M.K.); 2 Wissen Co., Ltd., #410 Bio Venture Town, 461-8, Daejeon 305-811, Korea; E-Mails: muzekokomi@hanmail.net (P.-M.H.); confessor@hanmail.net (J.-W.J.); jkypark@live.co.kr (J.-K.P.); 3 Department of Veterinary Internal Medicine, College of Veterinary Medicine, Chonnam National University, Gwangju 500-757, Korea; E-Mail: ghsuh@chonnam.ac.kr; 4 Department of Pulmonary Immunology, University of Texas Health Science Center at Tyler, 11937 US Hwy 271, Tyler, TX 75708-3154, USA

**Keywords:** honeybee venom (HBV), porcine reproductive and respiratory syndrome virus (PRRSV), immune enhancing effect, viral clearance effect

## Abstract

Porcine reproductive and respiratory syndrome (PRRS) is a chronic and immunosuppressive viral disease that is responsible for substantial economic losses for the swine industry. Honeybee venom (HBV) is known to possess several beneficial biological properties, particularly, immunomodulatory effects. Therefore, this study aimed at evaluating the effects of HBV on the immune response and viral clearance during the early stage of infection with porcine reproductive and respiratory syndrome virus (PRRSV) in pigs. HBV was administered via three routes of nasal, neck, and rectal and then the pigs were inoculated with PRRSV intranasally. The CD4^+^/CD8^+^ cell ratio and levels of interferon (IFN)-γ and interleukin (IL)-12 were significantly increased in the HBV-administered healthy pigs via nasal and rectal administration. In experimentally PRRSV-challenged pigs with virus, the viral genome load in the serum, lung, bronchial lymph nodes and tonsil was significantly decreased, as was the severity of interstitial pneumonia, in the nasal and rectal administration group. Furthermore, the levels of Th1 cytokines (IFN-γ and IL-12) were significantly increased, along with up-regulation of pro-inflammatory cytokines (TNF-α and IL-1β) with HBV administration. Thus, HBV administration—especially via the nasal or rectal route—could be a suitable strategy for immune enhancement and prevention of PRRSV infection in pigs.

## 1. Introduction

Honeybee (*Apis mellifera*) venom (HBV) has long been used as a therapeutic agent in alternative medicine for alleviation of pain, inflammation, and some immune system-related diseases such as rheumatoid arthritis and multiple sclerosis [1]. Moreover, accumulating evidence indicates that HBV exerts immunomodulatory effects on Th1 responses. Nam *et al*. [2] reported that whole HBV administration induces development of a Th1 lineage from the CD4^+^ T lymphocyte population and enhances the expression of interferon-gamma (IFN-γ) in a mouse model. In addition, Perrin-Cocon *et al*. [3] and Ramoner *et al*. [4] reported that a phospholipase in HBV induces the maturation of dendritic cells and activates dendritic-cell associated immune responses. Whole HBV contains at least 18 active ingredients, which include enzymes, biogenic amines, a protease inhibitor, and several biologically active peptides such as melittin, apamine, and adolapine [1]. Melittin, the principal component that is extracted from the water-soluble phase of HBV, has been reported to show multiple pharmacological effects, such as anti-microbial [5,6], anti-viral [7], and anti-cancer [8] properties. For this reason, many commercial products containing HBV components have been developed and manufactured with the emphasis on the melittin content as either the sole ingredient or a mixture of water-soluble components. On the other hand, the lipid-soluble portion of HBV has received less attention and has been usually neglected by the manufacturers of HBV. Some lipid-soluble components of HBV such as chrysin and pinocembrin-known as “flavonoids”-have been reported to show antiviral and antimicrobial effects [9,10]. Therefore, we developed a new HBV product that combines the lipid-soluble ingredients (chrysin and pinocembrin) with melittin to enhance the immunostimulatory and viral clearance effects of HBV. At present, it is uncertain whether immunostimulatory effect of HBV exists in pigs and whether HBV can be applied as an immunostimulatory agent for prevention of viral diseases in the swine industry. Nevertheless, there is growing evidence supporting the concept that HBV mainly enhances the CD4^+^/CD8^+^ T lymphocyte subset ratio, relative mRNA expression levels of IL-18 and IFN-γ and serum lysozyme activity, as shown in our previous study [11].

Porcine reproductive and respiratory syndrome (PRRS) is an economically important disease affecting the swine-producing industry in many countries [12]. This syndrome is characterized by reproductive failure in sows (such as abortions and stillbirths), increased pre-weaning mortality, induced respiratory disorders, and poor growth performance in growing pigs [13]. Infected pigs also have weak and delayed adaptive immune responses, and consequently, the susceptibility of such pigs to other viral infections and secondary bacterial infections is increased [14].

Thus, on the basis of our previous report, this study aimed at addressing several objectives: (a) evaluating the immunostimulatory efficacy of HBV in the pig immune system; (b) assessing the viral clearance activity of an HBV product containing melittin, chrysin and pinocembrin in pigs experimentally infected with PRRS virus (PRRSV; as an initial step towards the prevention of the viral diseases) and to elucidate the host immune responses, especially the pro-inflammatory response and changes in Th1-related cytokines, including IFN-γ, IL-12, TNF-α, and IL-1β (which are associated with protection from PRRSV); and (c) determining whether nasal, neck, and rectal administration of HBV induce comparable immune responses and viral clearance effects.

## 2. Results

### 2.1. Characteristics of HBV

**Figure 1:**
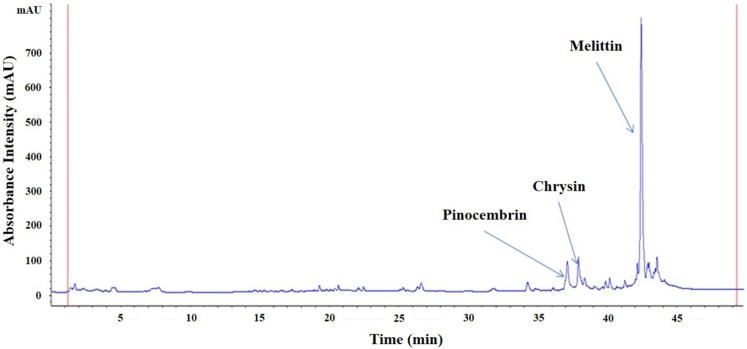
The high-performance liquid chromatography (HPLC) chemical fingerprint of honeybee venom (HBV) at 270 nm. Melittin, pinocembrin and chrysin were detected as components (mAU: arbitrary units).

The HBV components pinocembrin, chrysin and melittin were detected by high-performance liquid chromatography (HPLC). The retention times of pinocembrin, chrysin and melittin were 37, 38, and 42.5 min, respectively (Figure 1).

### 2.2. Effects of HBV on T Lymphocyte CD4^+^/CD8^+^ Subsets in Healthy Pigs

**Figure 2:**
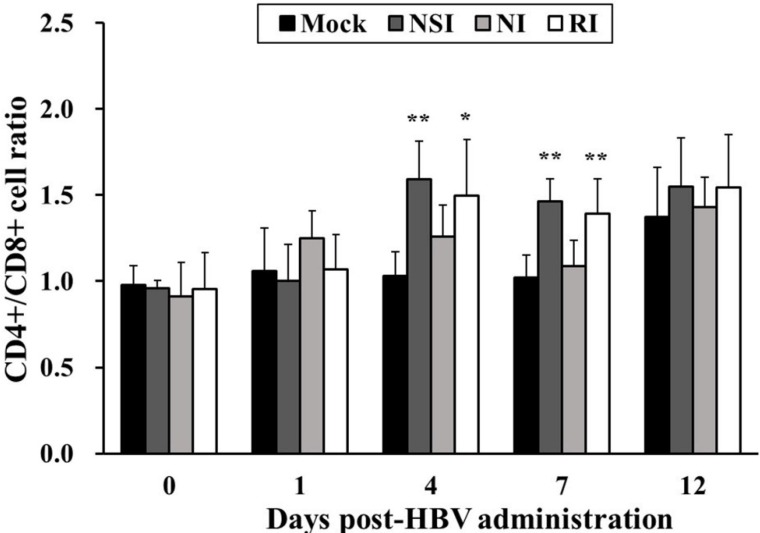
Effects of honeybee venom (HBV) on T lymphocyte CD4^+^/CD8^+^ subsets in the peripheral blood. After administration of HBV to pigs via three routes—nasal (NSI group), neck (NI group), and rectal (RI group)—the lymphocytes were isolated one day before HBV administration (0) and at 1, 4, 7 and 12 days post-HBV administration (DPA), and were analyzed for T lymphocyte CD4^+^/CD8^+^ subsets using flow cytometry. A higher CD4^+^/CD8^+^ T lymphocyte ratio was evident in the NSI and RI groups compared to the mock group at 4 and 7 DPA. The data are presented as mean ± SD of five pigs in each group. Significant differences with the mock group are presented as * *p* < 0.05 and ** *p* < 0.01.

The CD4^+^/CD8^+^ cell ratio in all HBV-administration groups tended to increase during the entire experimental period in comparison with the mock group (Figure 2). In particular, the difference in the CD4^+^/CD8^+^ cell ratio was significant between the mock group and groups “nasal injection” (NSI) and “rectal injection” (RI) at 4 (*p* < 0.05 for group RI and *p* < 0.01 for group NSI) and seven days post HBV-administration (DPA; *p* < 0.01).

### 2.3. Effects of HBV on Cytokine Expression Levels in Healthy Pigs

**Figure 3:**
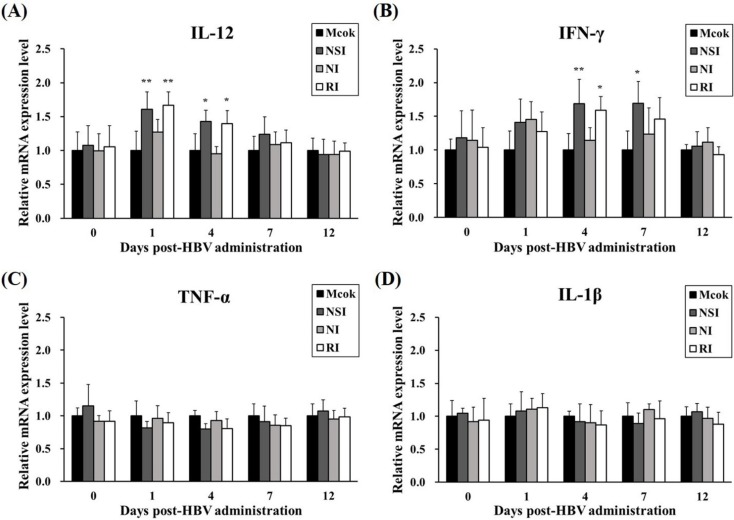
Effects of honeybee venom (HBV) on the mRNA expression levels of Th1 cytokines and pro-inflammatory cytokines. The expression levels of (**A**) IL-12, (**B**) IFN-γ, (**C**) TNF-α, and (**D**) IL-1β in peripheral blood mononuclear cells (PBMCs) were measured by quantitative real-time PCR and are presented after normalization to β-actin levels. The data are presented as mean ± SD of five pigs in each group. Significant differences with the mock group are presented as * *p* < 0.05 and ** *p* < 0.01. (NSI; nasal injection group, NI; neck injection group, and RI; rectal injection group).

The level of IL-12 significantly increased in HBV-administration groups at 1 DPA (*p* < 0.01 compared to the mock group) and at 4 DPA (*p* < 0.05 compared to the mock group; Figure 3A). Furthermore, the level of IFN-γ was significantly higher in group NSI at 4 and 7 DPA (*p* < 0.01 and *p* < 0.05, respectively) and group RI at 4 DPA (*p* < 0.05) in comparison with the mock group (Figure 3B). The relative expression levels of TNF-α and IL-1β (considered pro-inflammatory cytokines) did not show any statistically significant differences between the mock group and the HBV-administration groups (Figure 3C,D).

### 2.4. Effects of HBV on Viral Clearance in the PRRSV Infected Pigs

**Figure 4:**
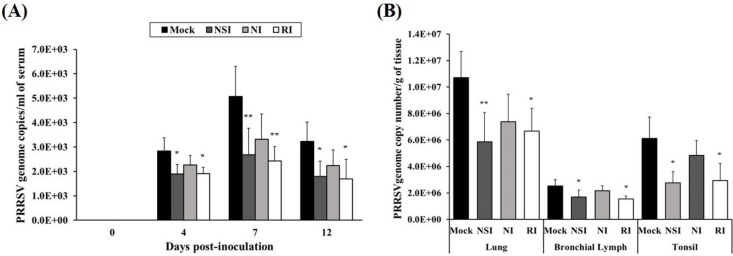
Effects of honeybee venom (HBV) on viral clearance of porcine reproductive and respiratory syndrome virus (PRRSV) in experimentally infected pigs. HBV was administered to pigs via three routes—nasal, neck, and rectal—followed by experimental PRRSV inoculation through both nostril. For quantification of the PRRSV genome, (**A**) serum was collected at 0, 4, 7, and 12 days post-inoculation (DPI), and (**B**) tissue samples (lung, bronchial lymph nodes and tonsils) were collected at post-mortem examination. The viral genome load in the serum samples was significantly decreased in groups NSI and RI compared to the mock group at 4, 7, and 12 DPI. In the tissue samples, groups NSI and RI showed a significant reduction in the viral load in the lung, bronchial lymph nodes (BLNs), and tonsil tissues, in comparison with the mock group. The data are expressed as mean ± SD of five pigs in each group. Significant differences with the mock group are presented as * *p* < 0.05, and ** *p* < 0.01. (NSI; nasal injection group, NI; neck injection group, and RI; rectal injection group).

The load of the PRRSV genome in serum was measured using real-time quantitative PCR and groups NSI and RI group showed a marked reduction in the load of the PRRSV genome in serum at 4, 7, and 12 days post-inoculation (DPI; *p* < 0.05 at 4 and 12 DPI, *p* < 0.01 at 7 DPI) compared to the mock group (Figure 4A). At the end of the experiment, lung, bronchial lymph nodes (BLNs) and tonsils were collected and analyzed for the load of the PRRSV genome. The viral genome load in lung tissue of groups NSI and RI showed a significant reduction (*p* < 0.05 for group RI and *p* < 0.01 for group NSI compared to the mock group). Similarly, the PRRSV load of BLNs and tonsil tissues was significantly decreased (*p* < 0.05) in groups NSI and RI compared to the mock group (Figure 4B).

### 2.5. Evaluation of Body Temperature and Histopathological Examination of the PRRSV Infected Pigs

**Figure 5:**
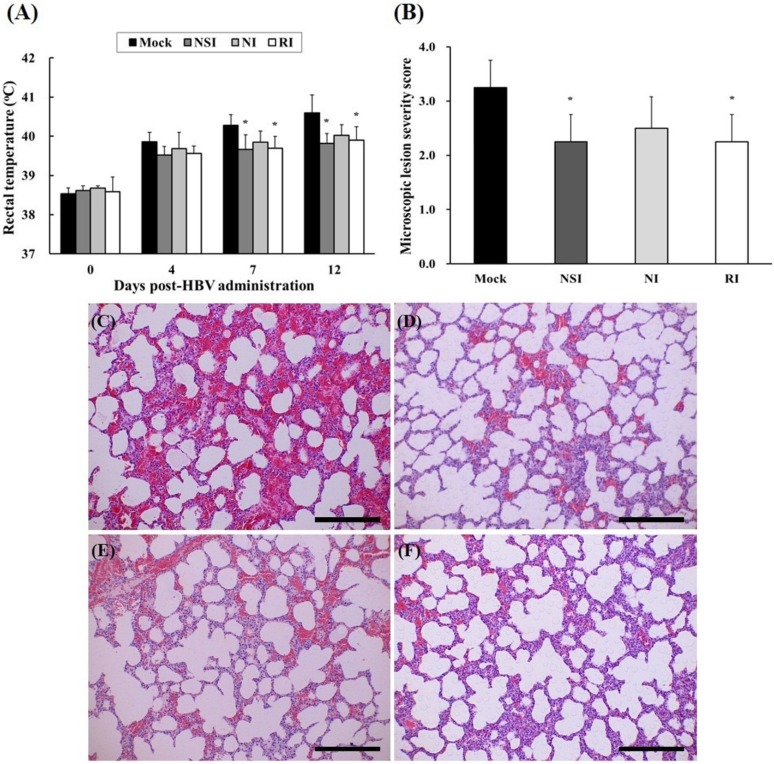
Changes of rectal temperature and histopathological evaluation of lung tissue in the pigs experimentally infected with porcine reproductive and respiratory syndrome virus (PRRSV). Rectal temperature was measured at 4, 7, and 12 DPI (**A**) and the lung tissue samples were analyzed by means of hematoxylin and eosin (H&E) staining in (**C**) the mock group, (**D**) group “nasal injection” (NSI), (**E**) group “neck injection” (NI), and (**F**) group “rectal injection” (RI) for evaluation of the severity of interstitial pneumonia. All pigs showed increased rectal temperature over 39.5 °C, and the groups NSI and RI showed a significant decrease in rectal temperature at 7 and 12 DPI in comparison with the mock group. Mild interstitial pneumonia was evident in the HBV-administration groups, and the lung microscopic lesion score (**B**) was significantly decreased in groups NSI and RI compared to the mock group (* *p* < 0.05). The data are expressed as mean ± SD of five pigs in each group. In all panels, magnification is at 200× and the scale bars are 100 μm.

After experimental infection with PRRSV, rectal temperature of all pigs rapidly increased to over 39.5 °C, and groups NSI and RI showed significant decrease compared to the mock group at 7 and 12 DPI (Figure 5A). The tissue lesions in the HBV-administration groups were milder compared to those of the mock group (Figure 5C–F). Especially, the differences were evident in the NSI and RI group compared with that of the mock group (*p* < 0.05; Figure 5B).

### 2.6. Effects of HBV on T Lymphocyte CD4^+^/CD8^+^ Subsets in the PRRSV Infected Pigs

**Figure 6:**
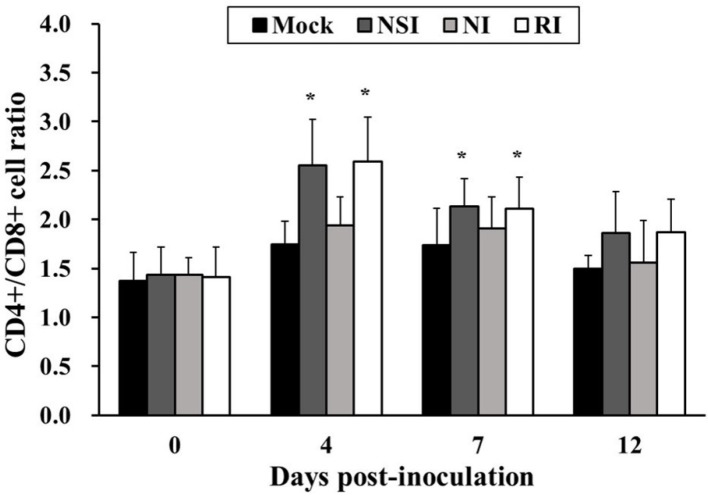
Effects of honeybee venom (HBV) on T lymphocyte CD4^+^/CD8^+^ subsets in the peripheral blood in pigs experimentally infected with porcine reproductive and respiratory syndrome (PRRSV). The PBMCs were isolated at 0, 4, 7, and 12 days post-inoculation (DPI) and were analyzed for the CD4^+^/CD8^+^ cell ratio by using flow cytometry. The CD4^+^/CD8^+^ T lymphocyte subset ratio was significantly increased in groups “nasal injection” (NSI) and “rectal injection” (RI) as compared to the mock group at 4 and 7 DPI. The data are presented as mean ± SD of five pigs in each group. Significant differences with the mock group are presented as * *p* < 0.05.

The CD4^+^/CD8^+^ cell ratio in groups NSI and RI tended to increase during the entire study period in comparison with the mock group. In particular, the difference in the CD4^+^/CD8^+^ cell ratio was significant between the mock group and groups NSI and RI at 4 and 7 DPI (*p* < 0.05; Figure 6).

### 2.7. Effects of HBV on Cytokine Expression Profiles in the PRRSV Infected Pigs

**Figure 7:**
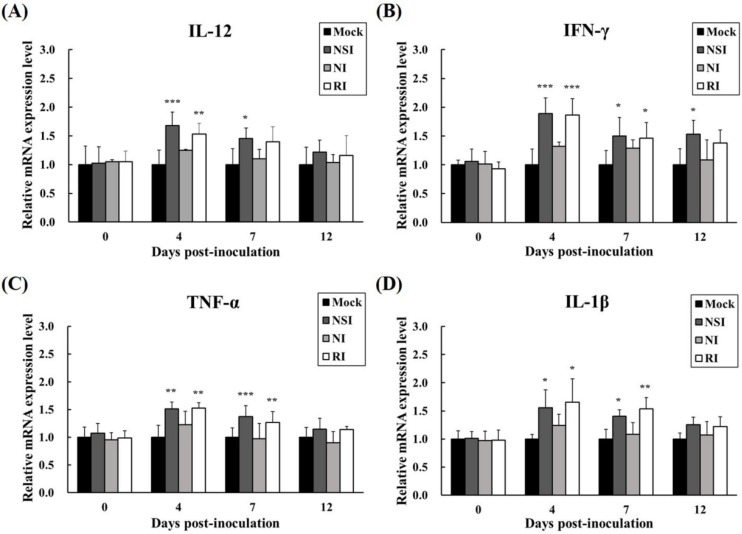
Effects of honeybee venom (HBV) on expression levels of Th1 and pro-inflammatory cytokines in experimentally PRRSV-infected pigs. The expression levels of (**A**) IL-12, (**B**) IFN-γ, (**C**) TNF-α, and (**D**) IL-1β were measured by means of quantitative real-time PCR and are presented after normalization to β-actin followed by the ΔΔCt method. The data are presented as mean ± SD of five pigs in each group. Significant differences with the mock group are presented as * *p* < 0.05, ** *p* < 0.01, and *** *p* < 0.001. (NSI; nasal injection group, NI; neck injection group, and RI; rectal injection group).

The relative mRNA expression levels of Th1 cytokines (IFN-γ and IL-12) and pro-inflammatory cytokines (TNF-α and IL-1β) were measured in PBMCs at the early stage of PRRSV infection (4, 7, and 12 DPI). All cytokines investigated showed similar increasing kinetic patterns (Figure 7). Especially, the level of IL-12 was significantly increased in groups NSI and RI at 4 DPI (*p* < 0.001 for group NSI and *p* < 0.01 for group RI compared to the mock group) and 7 DPI (*p* < 0.05 for group NSI compared to the mock group; Figure 7A). Similarly, IFN-γ levels were markedly increased in groups NSI and RI at 4 DPI (*p* < 0.001 for groups NSI and RI compared to the mock group), at 7 DPI (*p* < 0.05 for groups NSI and RI compared to the mock group), and at 12 DPI (*p* < 0.05 for group NSI compared to the mock group; Figure 7B). The pro-inflammatory cytokines also showed statistically significant differences between the mock group and groups NSI and RI. Notably, the level of TNF-α significantly increased at 4 DPI (*p* < 0.01 for groups NSI and RI compared to the mock group) and at 7 DPI (*p* < 0.001 for group NSI and *p* < 0.01 for group RI compared to the mock group; Figure 7C). The level of IL-1β also markedly increased at 4 DPI (*p* < 0.05 for groups NSI and RI compared to the mock group) and at 7 DPI (*p* < 0.05 for group NSI and *p* < 0.01 for group RI compared to the mock group; Figure 7D).

## 3. Discussion

In the present study, HBV was administered to pigs through three routes—nasal, neck, and rectal—and then the CD4^+^/CD8^+^ cell ratio and the expression levels of Th1 and pro-inflammatory cytokines were evaluated. The neck region is a common route for administration of classical vaccines in pigs. The nasal and rectal regions are newly highlighted sites for vaccine administration, and this approach can induce both mucosal and systemic immunity. The mucosal regions, which are classified into ocular, nasal, oral, pulmonary, and vaginal or rectal, comprise ~80% of all immune cells in the body [15]. Particulate antigens (such as microspheres, liposomes, and bacterial ghosts) that are delivered via a mucosal route are recognized by microfold cells (M cells) located in mucosa-associated lymphoid tissues and are then presented to professional antigen-presenting cells (APCs) such as dendritic cells and macrophages. Consequently, APCs forcefully stimulate differentiation of T cells and effectively induce specific adaptive immune responses [16]. In particular, optimal stimulation of mucosal immunity is effective for protection from many infectious diseases because it can stimulate both mucosal and systemic immunity, leading to inhibition of entry of pathogens into the body [15]. According to the present study, the CD4^+^/CD8^+^ cell ratio in all HBV-administration groups tends to increase during the entire experimental period. Notably, the change in the CD4^+^/CD8^+^ cell ratio is statistically significant in group RI at 4 and 7 DPA and in group NSI at 7 DPA. The number and ratio of two main T lymphocyte subsets, CD4^+^ cells (or T helpers) and CD8^+^ cells (or T cytotoxic) were used as the most meaningful parameters for evaluation of immune homeostasis and response of the intrinsic immune system [17]. Furthermore, low CD4^+^/CD8^+^ cell ratio are usually related to acute viral diseases and hemophilia, whereas high ratios are observed individually with stimulation of immuno-functional ability [11]. Simultaneously with the CD4^+^/CD8^+^ cell ratio, the level of Th1 cytokines (IL-12 and IFN-γ) is significantly increased in groups NSI and RI at 1, 4, and 7 DPA. These findings indicate that administration of HBV effectively induces Th1-related immune responses up to 7 DPA, and this phenomenon may correlate with the increasing CD4^+^/CD8^+^ cell ratio through stimulation of the CD4^+^ T cell population when HBV is administered via the nasal or rectal route. These results are consistent with other reports showing that HBV administration to broiler chicks (using a spray method) significantly increases INF-γ expression levels and the CD4^+^/CD8^+^ cell ratio [11].

PRRSV is a unique viral pathogen because this virus can evade host immune surveillance or effectively control both innate and adaptive immune regulators [18,19]. PRRSV-infected pigs typically show insufficient anti-PRRSV immunity because of low levels of Th1 cytokine (IFN-γ and IL-12) production, which are typically involved in cellular immunity [20]. IFN-γ is known to possess potent antiviral properties against PRRSV infection through the inhibition of viral replication and to be an important mediator of cellular immunity [21]. Furthermore, IL-12 plays a critical role in host immunity against viral infections through the promotion of IFN-γ production and Th1 cell type differentiation [19]. Therefore, the PRRSV-induced suppression of Th1-related cytokines may result in prolonged immunosuppression, followed by weaker antibody responses, increased levels of viremia, and greater severity of the disease [22]. Simultaneously with the repression of Th1 responses, PRRSV acts via unique mechanisms whereby the production of pro-inflammatory cytokines, such as TNF-α and IL-1β, is limited during the PRRSV infection, in contrast to other viral respiratory diseases of pigs [23]. TNF-α performs an important function in the inflammatory response and in the protection of the cells from viral infection or in promotion of selective elimination of virus-infected cells via IFN-independent mechanism [24]. The PRRSV-induced suppressive action on TNF-α and IL-1β production, as a strategy for modulation of host immune responses, thus contributes to the unique clinical features; the absence of a fever correlates with the development of more severe interstitial pneumonia after PRRSV infection [22]. Accordingly, there are reports about weak expression of pro-inflammatory cytokines (TNF-α and IL-1β) in pigs infected with PRRSV-1 and PRRSV-2 [14]. In the present study, levels of Th1 cytokines (IFN-γ and IL-12) and pro-inflammatory cytokines (TNF-α and IL-1 β) were significantly increased in groups NSI and RI, especially 4 and 7 DPI compared to the mock group. In addition, the PRRSV genome loads in serum and PRRSV-infected tissues (lung, BLNs and tonsils) were considerably reduced in HBV-administration groups, especially in groups NSI and RI. Similarly, the viral load of PRRSV-infected tissues is markedly reduced in groups NSI and RI. Additionally, histopathological analysis of the lung tissue revealed that groups NSI and RI undergo less pathological changes in comparison with the mock group. Pigs that are experimentally infected with PRRSV typically show mild respiratory and systemic clinical signs, where the absence of a fever correlates with more severe interstitial pneumonia [14]. Therefore, these findings suggest that HBV administration, especially via the nasal or rectal route, enhances clearance of PRRSV in experimentally infected pigs, and these viral clearance effect of HBV may be related to the up-regulation of Th1 and partial pro-inflammatory immune responses. These findings are similar to those by Dwivedi *et al*. [19], who found that vaccines consisting of the PLGA nanoparticle-entrapped killed PRRS virus exhibit high protective efficacy in pigs against a PRRSV challenge (in comparison with classically vaccinated pigs); specifically, there is significant up-regulation of Th1 cytokines (IFN-γ and IL-12) and several types of immune cells, including CD3^+^CD8^+^, CD4^+^CD8^+^, and γδ T cells.

In summary, the data from our results suggest that administration of HBV via a nasal or rectal route can effectively enhance the Th1-specific systemic immunity in healthy pigs and reduce the viral load and severity of interstitial pneumonia in PRRSV-infected pigs. These immunomodulatory effects and PRRSV clearance that are induced by the administration of HBV may be related to promotion of Th1-specific responses and inhibition of PRRSV-specific mechanisms that involve down-regulation of pro-inflammatory cytokines. Thus, our findings—in conjunction with further research on HBV in a field study (for confirmation of the protective efficacy against a naturally occurring PRRSV infection)—may lead to new immunostimulatory and preventive strategies for PRRSV infection in the swine industry.

## 4. Materials and Methods

### 4.1. Preparation and Characterization of HBV

Crude HBV was obtained using a Large Quantity Bee-Venom Collector (P10-1003672, Wissen, Daejeon, Korea). We extracted 2.5g of crude HBV with 250 mL of ultra-filtered water and ethyl acetate three times at room temperature (RT) and passed the extract through a filter containing a nylon membrane (0.45-μm pore diameter; Millipore, Billerica, MA, USA) under vacuum driven filtration system. The two filtrates were mixed and then concentrated in vacuum at 40 °C. The final HBV concentrates (10 mg) were dissolved in 1 mL of ultra-filtered water and analyzed by HPLC. The HBV samples that we used for HPLC analysis were passed through a 0.45-μm filter (Millipore) before injection into a UPLC^®^ BEH C18 column (1.7 µm, 2.1 × 100 mm; Waters Corporation, Milford, MA, USA). The gradient ratios of mobile phases A (0.1% trifluoroacetic acid in methanol) and B (0.1% trifluoroacetic acid in distilled water) were set at 0:100 for 0–10 min, with a flow rate of 0.2 mL/min, and then were set at 50:50 for 20–30 min until the last step, which were set at 100:0 at 50 min, with a flow rate of 0.3 mL/min. The wavelengths for detection was 270 nm. For the experimental use, the final HBV fine powder was dissolved in a solvent consisting of 95.7% distilled water, 3.5% ethanol, and 0.8% propylene glycol, by volume. The HBV concentration was 2.1 mg/mL, which was the optimal concentration according to our preliminary experiments.

### 4.2. PRRS Virus

The PRRSV type II LMY strain (GenBank accession number DQ473474), which was originally isolated in South Korea from a pig with naturally occurring PRRS. LMY strain was kindly provided by Prof. Dr. Kyoung-oh Cho (Chonnam National University, Gwangju, Korea). For preparation of the virus inoculum, the virus stock was propagated in confluent monolayers of MARC-145 cells, as described previously [21], and the virus titer was measured using quantitative real-time PCR.

### 4.3. Animals

Conventional 4-week-old pigs, cross-breeds between Landrace, Yorkshire and Duroc, were obtained from a single healthy herd without any history of PRRS. All pigs were housed in separate air-conditioned rooms and were allowed free access to nutritionally complete antibiotic-free pig feed (Daehan Livestock & Feed, Naju, Jeonnam, Korea) and drinking water. Prior to the experiment, all pigs were tested and confirmed to be seronegative for antibodies to PRRSV by using a commercial enzyme-linked immunosorbent assay kit (IDEXX Laboratories, Westbrook, ME, USA) and tested negative for presence of viremia by real-time PCR [25].

### 4.4. Experimental Protocol

#### 4.4.1. Experiment 1: Immunostimulatory Effects of HBV on Healthy Pigs

The pigs were randomly allocated to four groups of five pigs each and were injected with HBV subcutaneously via different routes as follows: Group 1, mock control pigs; Group 2, injected with 2.1 mg of HBV into the nasal region (NSI group); Group 3, injected with 2.1 mg of HBV into the neck region (NI group); Group 4, injected with 2.1 mg of HBV into the rectal region (RI group). To measure the immunostimulatory effect of HBV, blood samples were individually collected from the jugular vein 1 day before HBV administration and at 1, 4, 7 and 12 DPA. Body weight was also monitored during the whole study period. All animal procedures were performed in accordance with the guidelines of International Guiding Principles for Biomedical Research Involving Animals by the Council for International Organizations of Medical Sciences. (CIOMS, care of the World Health Organization, Geneva, Switzerland) and approved by the Institutional Animal Care and Use Committee of Chonnam National University (approval number: CNU IACUC-YB-2013-29).

#### 4.4.2. Experiment 2: The Viral Clearance Effect of HBV in the Pigs Experimentally Infected with PRRSV

The pigs were randomly subdivided into groups and injected with HBV, as described in Experiment 1. After 4 days of HBV administration, five milliliters of the viral culture (1 × 10^6^ copies/mL, optimized previously) was inoculated intranasally into both nostril of each pig. After PRRSV infection, blood samples were collected at 0, 4, 7, and 12 DPI for analysis of T lymphocyte CD4^+^/CD8^+^ subsets and cytokine expression profiles. Clinical symptoms and rectal temperature were also monitored every day during the entire study period (12 days), as described previously [23]. At the end of the experiment, all pigs in all groups were euthanized for collection of lung and lymphoid tissues (BLNs and tonsils) for titration of the virus.

### 4.5. Isolation of PBMCs from Peripheral Blood

PBMCs were isolated as described previously [11]. Briefly, blood samples were collected into lithium-heparin coated tubes and diluted with equal volumes of phosphate buffered saline (PBS). The diluted mixture was layered over a half of the volume of Lymphoprep (Axis-shield, Oslo, Norway) and was separated by gradient centrifugation at 800 × *g* for 30 min at RT. Contaminating red blood cells were lysed using commercial Red Blood Cell Lysing Buffer Hybri-Max (Sigma-Aldrich, St. Louis, MO, USA). The PBMCs were then washed twice with PBS prior to resuspension in a complete medium consisting of RPMI-1640 medium (Lonza, Basel, Switzerland) containing 10% (*v*/*v*) fetal bovine serum (FBS; Gibco, Grand Island, NY, USA) and 2% (*v*/*v*) of the antibiotic-antimycotics formulating solution with penicillin, streptomycin, and amphotericin B (Lonza, Basel, Switzerland).

### 4.6. Analysis of T Lymphocyte CD4^+^/CD8^+^ Subsets by using Flow Cytometry

The PBMCs obtained from the peripheral blood were analyzed to determine the CD4^+^CD8^−^ T lymphocyte and CD4^−^CD8^+^ T lymphocyte subset ratio within the CD3^+^ subset by means of flow cytometry as described previously [19]. In brief, the cells were stained with a fluorescein isothiocyanate (FITC)-conjugated mouse anti-pig CD3ε antibody (clone BB23-8E6-8C8; BD Biosciences, Franklin Lakes, NJ, USA), a phycoerythrin (PE)/Cy7-conjugated mouse anti-pig CD4a antibody (clone 74-12-4; BD Biosciences, Franklin Lakes, NJ, USA), and a PE-conjugated mouse anti-pig CD8a antibody (clone 76-2-11; BD Biosciences, Franklin Lakes, NJ, USA). After incubation at RT for 30 min in the dark, the cells were washed twice with PBS and the lymphocyte subpopulations were analyzed using a BD Accuri C6 flow cytometer (BD Biosciences, Franklin Lakes, NJ, USA).

### 4.7. Analysis of Cytokine Expression Levels in PBMCs

We measured the expression levels of IFN-γ, IL-12, TNF-α, and IL-1β in the PBMCs to evaluate the immunostimulatory effect of HBV, especially that related to the pro-inflammatory and Th1 cytokines in the pig immune system. Total RNA was extracted from the PBMCs by using the commercial PureLink RNA Mini Kit (Invitrogen, Grand Island, NY, USA). The RNA concentration was quantified using a NanoDrop ND-1000 instrument (Thermo Fisher Scientific, Waltham, MA, USA), and equal amounts of RNA were reverse-transcribed using the QuantiTect Reverse Transcription Kit (Qiagen, Valencia, CA, USA). To minimize variation in the reverse-transcriptase efficiency, all samples were transcribed simultaneously. Quantitative real-time PCR was performed using a MyiQ2 thermocycler and the SYBR green detection system (Bio-Rad Laboratories, Hercules, CA, USA). The real-time PCR conditions were as follows: 95 °C for 10 min, then 45 cycles at 95 °C, 30 s; 57 °C, 30 s; and 72 °C, 30 s. The oligonucleotide primer pairs that we used for analysis of pig IFN-γ, IL-12, TNF-α, IL-1β, and β-actin expression are shown in Table 1. We confirmed that reaction efficiencies of all primers range between 95% and 105% and R^2^ value is over 0.98. The threshold cycle was then determined subsequently. Relative quantitation of pig IFN-γ, IL-12, TNF-α, and IL-1β expression levels were calculated using the comparative Ct method as described previously [26]. The relative quantitation value of the target gene was normalized to an endogenous control β-actin gene, and the differences between control and HBV groups were evaluated as 2^−ΔΔCt^ method (fold change).

**Table 1:** The real-time PCR primer sequences

Gene	Sequence (5'–3')		Accession number
TNF-α	Forward	CCCCCAGAAGGAAGAGTTTC	JF831365
	Reverse	CGGGCTTATCTGAGGTTTGA	
IL-1β	Forward	GGCCGCCAAGATATAACTGA	NM_214055
	Reverse	GGACCTCTGGGTATGGCTTTC	
IFN-γ	Forward	CAAAGCCATCAGTGAACTCATCA	X53085
	Reverse	TCTCTGGCCTTGGAACATAGTCT	
IL-12	Forward	GGAGTATAAGAAGTACAGAGTGG	U08317
	Reverse	GATGTCCCTGATGAAGAAGC	
β-actin	Forward	CAGGTCATCACCATCGGCAACG	U07786
	Reverse	GACAGCACCGTGTTGGCGTAGAGGT	

### 4.8. Quantification of PRRSV Genome in Serum and Tissues using Quantitative Real-Time PCR

The serum samples were prepared from the blood samples at 0, 4, 7, and 12 DPI and were collected in EDTA-free tubes after the removal of clots and centrifugation. Lung and lymphoid tissues were homogenized (10%, *w*/*v*) in minimum essential medium alpha modification (alpha-MEM; Lonza, Basel, Switzerland) containing 2% (*v*/*v*) of an antibiotic-antimycotics solution consisting of penicillin, streptomycin, and amphotericin B (Lonza, Basel, Switzerland), and were then centrifuged at 4000 × *g* for 30 min at 4 °C. The supernatant was passed through a 0.20-µm non-pyrogenic syringe filter (Pall Corporation, Ann Arbor, MI, USA). RNA extraction from the collected supernatants was performed using the PureLink Viral RNA/DNA Mini Kit (Invitrogen, Grand Island, NY, USA) according to the manufacturer’s instructions, and equal amounts of targeted RNA was reverse transcribed into complementary DNA (cDNA) using the QuantiTect reverse transcription kit (Qiagen, Valencia, CA, USA). The cDNA was used for quantification of PRRSV genome by optimized real-time PCR reaction using a standard curve as previously described [27]. Briefly, for the construction of PRRSV genome plasmid, the ORF7 gene of PRRSV was amplified with the following primers: PRRSV forward, 5'-ATAACAACGGCAAGCAG-3'; PRRSV reverse, 5'-CAGTGTAACTTATCCTCCCA-3' (Genebank accession number: AF121131). The resulting PCR product was cloned into the pGEM-T Easy vector (Promega, Madison, WI, USA), which was then transformed into Escherichia coli JM109 competent cells. The plasmid DNA was purified using a Qiaprep Spin Miniprep Kit (Qiagen, Valencia, CA, USA) and quantified using a NanoDrop ND-1000 (Thermo Fisher Scientific, Waltham, MA, USA). According to the above-mentioned quantitation standard curve, real-time PCR for quantitation of the PRRSV genome was performed on a MyiQ2 thermocycler by using the SYBR green detection system (Bio-Rad Laboratories, Hercules, CA, USA). For each assay, a standard curve was constructed using serially diluted plasmid standards with PRRSV at 10^2^–10^9^ copy numbers/mL. The amplification condition were as follows: 10 min at 95 °C, followed by 50 cycles at 95 °C for 5 s, 57 °C for 15 s and 72 °C for 10 s.

### 4.9. Histopathological Analysis in Lung Tissue of the Pigs Experimentally Infected with PRRSV

At post-mortem examination, the lung tissue was fixed in 10% neutral-buffered formalin for histopathological examination. The fixed samples were routinely processed, embedded in paraffin, sectioned (at 5 µm thickness), and stained with H&E. Microscopic lesions in the lung tissue were examined by an unbiased certified veterinary pathologist, using previously described scoring systems [28,29] in a blinded fashion. The lung sections were scored for the severity of interstitial pneumonia, ranging from 0–4: 0 = no microscopic lesions; 1 = mild interstitial pneumonia; 2 = moderate multifocal interstitial pneumonia; 3 = moderate diffuse interstitial pneumonia; and 4 = severe interstitial pneumonia.

### 4.10. Statistical Analysis

The data were expressed as mean ± SD and the means of different parameters were compared among the groups by using one-way analysis of variance (ANOVA) followed by the Tukey-Kramer multiple comparison. All calculations were performed in the GraphPad InStat software, version 3.0 (GraphPad Software, La Jolla, CA, USA). Particularly, the Mann-Whitney U test (nonparametric test; SPSS software, version 17.0; SPSS, Chicago, IL, USA) was used to analyze differences in the microscopic-lesion score (ordinal data) in tissues of the pigs experimentally infected with PRRSV. *p* value <0.05 was assumed to indicate statistical significance.

## References

[1] Oršolić N. (2012). Bee venom in cancer therapy. Cancer Metastasis Rev..

[2] Nam S., Ko E., Park S.K., Ko S., Jun C.Y., Shin M.K., Hong M.C., Bae H. (2005). Bee venom modulates murine Th1/Th2 lineage development. Int. Immunopharmacol..

[3] Perrin-Cocon L., Agaugué S., Coutant F., Masurel A., Bezzine S., Lambeau G., André P., Lotteau V. (2004). Secretory phospholipase A2 induces dendritic cell maturation. Eur. J. Immunol..

[4] Ramoner R., Putz T., Gander H., Rahm A., Bartsch G., Schaber C., Thurnher M. (2005). Dendritic-cell activation by secretory phospholipase A2. Blood.

[5] Mataraci E., Dosler S. (2012). *In vitro* activities of antibiotics and antimicrobial cationic peptides alone and in combination against methicillin-resistant *Staphylococcus aureus* biofilms. Antimicrob. Agents Chemother..

[6] Liu H., Han Y., Fu H., Liu M., Wu J., Chen X., Zhang S., Chen Y. (2013). Construction and expression of sTRAIL-melittin combining enhanced anticancer activity with antibacterial activity in *Escherichia coli*. Appl. Microbiol. Biotechnol..

[7] Falco A., Barrajón-Catalán E., Menéndez-Gutiérrez M.P., Coll J., Micol V., Estepa A. (2013). Melittin-loaded immunoliposomes against viral surface proteins, a new approach to antiviral therapy. Antivir. Res..

[8] Son D.J., Lee J.W., Lee Y.H., Song H.S., Lee C.K., Hong J.T. (2007). Therapeutic application of anti-arthritis, pain-releasing, and anti-cancer effects of bee venom and its constituent compounds. Pharmacol. Ther..

[9] Schnitzler P., Neuner A., Nolkemper S., Zundel C., Nowack H., Sensch K.H., Reichling J. (2010). Antiviral activity and mode of action of propolis extracts and selected compounds. Phytother. Res..

[10] Rasul A., Millimouno F.M., Ali Eltayb W., Ali M., Li J., Li X. (2013). Pinocembrin: A novel natural compound with versatile pharmacological and biological activities. Biomed. Res. Int..

[11] Jung B.G., Lee J.A., Park S.B., Hyun P.M., Park J.K., Suh G.H., Lee B.J. (2013). Immunoprophylactic effects of administering honeybee (*Apis melifera*) venom spray against *Salmonella* Gallinarum in broiler chicks. J. Vet. Med. Sci..

[12] Nuemann E.J., Kliebenstein J.B., Johnson C.D., Mabry J.W., Bush E.J., Seitzinger A.H., Green A.L., Zimmerman J.J. (2005). Assessment of the economic impact of porcine reproductive and respiratory syndrome on swine production in the United States. JAVMA.

[13] Opriessnig T., Giménez-Lirola L.G., Halbur P.G. (2011). Polymicrobial respiratory disease in pigs. Anim. Health. Res. Rev..

[14] Gómez-Laguna J., Salguero F.J., Pallarés F.J., Carrasco L. (2013). Immunopathogenesis of porcine reproductive and respiratory syndrome in the respiratory tract of pigs. Vet. J..

[15] Holmgren J., Czerkinsky C. (2005). Mucosal immunity and vaccines. Nat. Med..

[16] Renukaradhya G.J., Dwivedi V., Manickam C., Binjawadagi B., Benfield D. (2012). Mucosal vaccines to prevent porcine reproductive and respiratory syndrome: a new perspective. Anim. Health. Res. Rev..

[17] Xu M., Zhao M., Yang R., Zhang Z., Li Y., Wang J. (2013). Effect of dietary nucleotides on immune function in Balb/C mice. Int. Immunopharmacol..

[18] Dwivedi V., Manickam C., Binjawadagi B., Linhares D., Murtaugh M.P., Renukaradhya G.J. (2012). Evaluation of immune responses to porcine reproductive and respiratory syndrome virus in pigs during early stage of infection under farm conditions. Virol. J..

[19] Dwivedi V., Manickam C., Binjawadagi B., Renukaradhya G.J. (2013). PLGA nanoparticle entrapped killed porcine reproductive and respiratory syndrome virus vaccine helps in viral clearance in pigs. Vet. Microbiol..

[20] Barranco I., Gómez-Laguna J., Rodríguez-Gómez I.M., Quereda J.J., Salguero F.J., Pallarés F.J., Carrasco L. (2012). Immunohistochemical expression of IL-12, IL-10, IFN-α and IFN-γ in lymphoid organs of porcine reproductive and respiratory syndrome virus-infected pigs. Vet. Immunol. Immunopathol..

[21] Charerntantanakul W., Yamkanchoo S., Kasinrerk W. (2013). Plasmids expressing porcine interferon gamma up-regulate pro-inflammatory cytokine and co-stimulatory molecule expression which are suppressed by porcine reproductive and respiratory syndrome virus. Vet. Immunol. Immunopathol..

[22] Shi K.C., Guo X., Ge X.N., Liu Q., Yang H.C. (2010). Cytokine mRNA expression profiles in peripheral blood mononuclear cells from piglets experimentally co-infected with porcine reproductive and respiratory syndrome virus and porcine circovirus type 2. Vet. Microbiol..

[23] Gómez-Laguna J., Salguero F.J., Barranco I., Pallarés F.J., Rodríguez-Gómez I.M., Bernabé A., Carrasco L. (2010). Cytokine expression by macrophages in the lung of pigs infected with the porcine reproductive and respiratory syndrome virus. J. Comp. Pathol..

[24] Gimeno M., Darwich L., Díaz I., de la Torre E., Pujols J., Martín M., Inumaru S., Cano E., Domingo M., Montoya M. (2011). Cytokine profiles and phenotype regulation of antigen presenting cells by genotype-I porcine reproductive and respiratory syndrome virus isolates. Vet. Res..

[25] Olvera A., Sibila M., Calsamiglia M., Segalés J., Domingo M. (2004). Comparison of porcine circovirus type 2 load in serum quantified by a real time PCR in postweaning multisystemic wasting syndrome and porcine dermatitis and nephropathy syndrome naturally affected pigs. J. Virol. Method..

[26] Livak K.J., Schmittgen T.D. (2001). Analysis of relative gene expression data using real-time quantitative PCR and the 2^−ΔΔCt^ Method. Methods.

[27] Chung W.B., Chan W.H., Chaung H.C., Lien Y., Wu C.C., Huang Y.L. (2005). Real-time PCR for quantitation of porcine reproductive and respiratory syndrome virus and porcine circovirus type 2 in naturally-infected and challenged pigs. J. Virol. Method..

[28] Opriessnig T., Thacker E.L., Yu S., Fenaux M., Meng X.J., Halbur P.G. (2004). Experimental reproduction of postweaning multisystemic wasting syndrome in pigs by dual infection with *Mycoplasma hyopneumoniae* and porcine circovirus type 2. Vet. Pathol..

[29] Manickam C., Dwivedi V., Patterson R., Papenfuss T., Renukaradhya G.J. (2013). Porcine reproductive and respiratory syndrome virus induces pronounced immune modulatory responses at mucosal tissues in the parental vaccine strain VR2332 infected pigs. Vet. Microbiol..

